# Supplementary material for: Investigating the Relationship between Home Parenteral Support and Needs-Based Quality of Life in Patients with Chronic Intestinal Failure: A National Multi-Centre Longitudinal Cohort Study
Source: Nutrients. 2023 Jan 25;15(3):622. doi: 10.3390/nu15030622 (PMC9921538; doi:10.3390/nu15030622)
Supplement: Supplementary file 1 [file nutrients-15-00622-s001.zip › nutrients-2139742-supplementary.pdf]

Table S1: Age and gender characteristics within the total population and the study sample population at each recruitment site

		Age		Gender			
**Population**	**N**	**M (*n*)**	**Mean (SD) yrs**	**M (*n*)**	**Male** *n* **(%)**	**Female** *n* **(%)**	**TG/Other** *n***(%)**
Salford							
Total	251	-	58.5 (15.4)	-	91 (36)	160 (64)	0
Study sample	125	-	60.28 (14.79)	-	50 (40)	75 (60)	0
London NW							
Total		-	54.4 (17.2)	-	137 (36.7)	234 (63)	1 (0.3)
Study	84	-	58.98 (17.38)	-	36 (43)	47 (56)	1 (1)
Leeds							
Total	195	-	58 (16)	-	72 (37)	123 (63)	0
Study sample	56	-	60.52 (15.37)	-	20 (36)	36 (64)	0
Nottingham							
Total	99	-	37.3 (13.5)	-	28 (28)	71 (72)	0
Study sample	31	1	64.47 (14.04)	-	12 (39)	19 (61)	0
West Midlands[a]							
Total	167	-	55.5 (15.95)	-	64 (38)	103 (62)	0
Study sample	59	2	54.42 (17.44)	2	21 (37)	36 (63)	0
Cambridge							
Total	160	-	53.2 (17.9)	-	55 (34)	105 (66)	0
Study sample	42	-	58.02 (18.60)	-	14 (33)	28 (67)	0
South[b]							
Total	138	-	55.5 (15.67)	-	54 (39)	84 (61)	0
Study sample	30	1	63.9 (12.9)	1	10 (34)	19 (66)	0
Glasgow							
Total	97	-	59.00 (13)	-	28 (29)	69 (71)	0
Study sample	28	1		1	14 (52)	13 (48)	0
Sheffield							
Total	50	-	58.9 (15.8)	-	15 (30)	35 (70)	0
Study sample	27	-	64.26 (11.68)	-	11 (41)	16 (59)	0
Leicester							
Total	53	-	56.8 (14.2)	-	26 (49)	27 (51)	0
Study sample	22	-	62.41 (11.60)	-	10 (45)	12 (55)	0

[a] Includes Birmingham and Coventry NHS sites

[b] Includes St Georges, Exeter and Norfolk NHS sites

N: number for total sample, *n*: number for a particular variable, M: missing data, SD: Standard deviation, TG: Transgender.
